# Supplementary material for: Efficiently handling constraints in mixed-integer nonlinear programming problems using gradient-based repair differential evolution
Source: PeerJ Comput Sci. 2024 May 31;10:e2095. doi: 10.7717/peerj-cs.2095 (PMC11157599; doi:10.7717/peerj-cs.2095)
Supplement: Supplemental Information 1 [file peerj-cs-10-2095-s001.pdf]

Supplemental File for "Efficiently Handling Constraints in Mixed-Integer Nonlinear Programming Problems Using Gradient-Based Repair Differential Evolution"

S-I. TEST PROBLEMS

F1 : minimize: $f(x,y) = (x-1)^2 + (y-3)^2$
subject to:

$$g(x,y) = (x+1)^2 + (y+1)^2 - 1 \leq 0$$
$$x \in [-3,1]$$
$$y \in \{-3,-2,-1,0,1\}$$

The optimal solution is x^*=-1 and y^*= 0, and $f(x^*, y^*)$ = 13.0000.

F2 : minimize: $f(x,\mathbf{y}) = x^2 + (y_1-1)^2 + (y_2-2)^2$
subject to:

$$g(x,\mathbf{y}) = x^2 + y_1^2 + 0.5y_2^2 - 1.5 \leq 0$$
$$x \in [-1,100]$$
$$y_1, y_2 \in \{-1,0,...,100\}$$

The optimal solution is x^*= 0 and $\mathbf{y}^*$= (1,1), and $f(x^*, \mathbf{y}^*)$ = 1.0000.

F3 : minimize: $f(x,y) = -x-y$
subject to:

$$g_1(x,y) = -x+y-2.005 \leq 0$$
$$g_2(x,y) = x-y+0.5 \leq 0$$
$$g_3(x,y) = 0.505x+y-3.505 \leq 0$$
$$x \in [-1,100]$$
$$y \in \{-1,0,...,100\}$$

The optimal solution is x^*= 1 and y^*= 3, and $f(x^*, y^*)$ = -4.0000.

F4 : minimize: $f(x,y) = -x-y$
subject to:

$$g_1(x,y) = y-3.4 \leq 0$$
$$g_2(x,y) = x-y \leq 0$$
$$x \in [-1,100]$$
$$y \in \{-1,0,...,100\}$$

The optimal solution is x^*= 3 and y^*= 3, and $f(x^*, y^*)$ = -6.0000.

F5 : minimize: $f(x,y) = (x-0.5)^2 + (y-1)^2$

subject to:

$$h(x,y) = -x^2 + y = 0$$
$$x \in [-1, 3.1]$$
$$y \in \{-1, 0, ..., 4\}$$

The best known optimal solution is x^*= 1 and y^*= 1, and $f(x^*, y^*)$ = 0.2500.

F6 : minimize: $f(x,y) = (x-10)^3 + (y-20)^3$

subject to:

$$g_1(x,y) = -(x-5)^2 - (y-4.86)^2 + 100 \leq 0$$
$$g_2(x,y) = (x-8)^2 + (y-5.48)^2 - 60 \leq 0$$
$$x \in [-1, 100]$$
$$y \in \{-1, 0, ..., 100\}$$

The best known optimal solution is x^*= 14.22498780 and y^*= 1, and $f(x^*, y^*)$ = -6783.5818.

F7 : minimize: $f(\mathbf{x},\mathbf{y}) = exp(x_1 x_2 x_3 y_1 y_2)$

subject to:

$$h_1(\mathbf{x},\mathbf{y}) = x_1^2 + x_2^2 + x_3^2 + y_1^2 + y_2^2 - 10 = 0$$
$$h_2(\mathbf{x},\mathbf{y}) = x_2 y_1 - 5 x_3 y_2 = 0$$
$$h_3(\mathbf{x},\mathbf{y}) = x_1^3 + y_1^3 + 1 = 0$$
$$x_1 \in [-2.3, 2.3]$$
$$x_2, x_3 \in [-3.2, 3.2]$$
$$y_1 \in \{-2, -1, 0, 1, 2\}$$
$$y_2 \in \{-3, -2, -1, 0, 1, 2, 3\}$$

The best known optimal solution is $\mathbf{x}^*$= (−1.25994205, −2.48314049, 0.496648098) and $\mathbf{y}^*$= (1, −1), and $f(\mathbf{x}^*, \mathbf{y}^*)$ = 0.2114.

F8 : minimize: $f(\mathbf{x},\mathbf{y}) = x_1 + x_2 + y_1$

subject to:

$$g_1(\mathbf{x},\mathbf{y}) = -1 + 0.0025(x_3 + x_4) \leq 0$$
$$g_2(\mathbf{x},\mathbf{y}) = -1 + 0.0025(-x_3 + y_2 + y_3) \leq 0$$
$$g_3(\mathbf{x},\mathbf{y}) = -1 + 0.01(x_5 - y_2) \leq 0$$
$$g_4(\mathbf{x},\mathbf{y}) = -x_1 x_4 + 833.33252 x_3 + 100 x_1 - 83333.333 \leq 0$$
$$g_5(\mathbf{x},\mathbf{y}) = -y_1 y_3 + 1250 y_2 + x_3 y_1 - 1250 x_3 \leq 0$$
$$g_6(\mathbf{x},\mathbf{y}) = -x_2 x_5 + 1250000 + x_2 y_2 - 2500 y_2 \leq 0$$
$$x_1 \in [100, 10000]$$
$$x_2 \in [1000, 10000]$$
$$x_3, x_4, x_5 \in [10, 1000]$$
$$y_1 \in \{1000, 1020, ..., 10000\}$$
$$y_2 . y_3 \in \{20, 40, ..., 1000\}$$

The best known optimal solution is $\mathbf{x}^*$= (555.55433833, 5000, 180, 220, 400) and $\mathbf{y}^*$ = (1500, 300, 280), and $f(\mathbf{x}^*, \mathbf{y}^*)$ = 7055.5544.

F9 : minimize: $f(\mathbf{x},\mathbf{y}) = x_1 + x_2 + y_1$

subject to:

$$
\begin{aligned}
& g_1(\mathbf{x},\mathbf{y}) = -1 + 0.0025(x_3 + x_4) \le 0 \\
& g_2(\mathbf{x},\mathbf{y}) = -1 + 0.0025(-x_3 + y_2 + y_3) \le 0 \\
& g_3(\mathbf{x},\mathbf{y}) = -1 + 0.01(x_5 - y_2) \le 0 \\
& g_4(\mathbf{x},\mathbf{y}) = -x_1x_4 + 833.33252x_3 + 100x_1 - 83333.333 \le 0 \\
& g_5(\mathbf{x},\mathbf{y}) = -y_1y_3 + 1250y_2 + x_3y_1 - 1250x_3 \le 0 \\
& g_6(\mathbf{x},\mathbf{y}) = -x_2x_5 + 1250000 + x_2y_2 - 2500y_2 \le 0 \\
& x_1 \in [100,10000], x_2 \in [1000,10000] \\
& x_3, x_4, x_5 \in [10,1000] \\
& y_1 \in \{1000,1050,...,10000\} \\
& y_2.y_3 \in \{50,100,...,1000\}
\end{aligned}
$$

The best known optimal solution is $\mathbf{x}^*$= (833.33171, 5000, 200, 200, 400) and $\mathbf{y}^*$= (1250, 300, 300), and $f(\mathbf{x}^*, \mathbf{y}^*)$ = 7083.3317.

F10 : minimize: $f(\mathbf{x},\mathbf{y}) = x_1 + x_2 + y_1$

subject to:

$$
\begin{aligned}
& g_1(\mathbf{x},\mathbf{y}) = -1 + 0.0025(x_3 + x_4) \le 0 \\
& g_2(\mathbf{x},\mathbf{y}) = -1 + 0.0025(-x_3 + y_2 + y_3) \le 0 \\
& g_3(\mathbf{x},\mathbf{y}) = -1 + 0.01(x_5 - y_2) \le 0 \\
& g_4(\mathbf{x},\mathbf{y}) = -x_1x_4 + 833.33252x_3 + 100x_1 - 83333.333 \le 0 \\
& g_5(\mathbf{x},\mathbf{y}) = -y_1y_3 + 1250y_2 + x_3y_1 - 1250x_3 \le 0 \\
& g_6(\mathbf{x},\mathbf{y}) = -x_2x_5 + 1250000 + x_2y_2 - 2500y_2 \le 0 \\
& x_1 \in [100,10000], x_2 \in [1000,10000] \\
& x_3, x_4, x_5 \in [10,1000] \\
& y_1 \in \{1000,1100,...,10000\} \\
& y_2, y_3 \in \{100,200,...,1000\}
\end{aligned}
$$

The best known optimal solution is $\mathbf{x}^*$= (833.33171, 5000, 200, 200, 400) and $\mathbf{y}^*$= (1300, 300, 300), and $f(\mathbf{x}^*, \mathbf{y}^*)$ = 7133.3317.

F11 : minimize: $f(\mathbf{x}) = \sum_{j=1}^{5}\sum_{i=1}^{5} c_{ij}x_{(10+i)}x_{(10+j)} + 2\sum_{j=1}^{5} d_j x^3_{(10+j)} - \sum_{i=1}^{10} b_i x_i$

subject to:

$$
\begin{aligned}
& g_j(\mathbf{x}) = -2\sum_{i=1}^{5} c_{ij}x_{(10+i)} - 3d_j x^2_{(10+j)} - e_j + \sum_{i=1}^{10} a_{ij}x_i \le 0, \\
& \qquad j = 1, \ldots, 5 \\
& x_1, x_2, x_4, x_6, ..., x_{11}, x_{13}, x_{14}, x_{15} \in [0,10] \\
& x_3, x_5, x_{12} \in \{0,1,...,10\}
\end{aligned}
$$

where $\mathbf{b}$ = [−40, −2, −0.25, −4, −4, −1, −40, −60, 5, 1]. The other data can be found in Table S-I. The best known optimal solution is $\mathbf{x}^*$= (x_1, x_2, x_4, x_6, . . . , x_{11}, x_{13}, x_{14}, x_{15}) = (0, 0, 0, 9.99999985, 0, 0,

0, 0, 0.28879805, 0.43951302, 0.31935496, 0.44885950) and $\mathbf{y}^*$= (x_3, x_5, x_{12}) = (4, 4, 0), and $f(\mathbf{x}^*, \mathbf{y}^*)$ = 33.5066.

$$\mathbf{F12}: \text{minimize: } f(\mathbf{x}) = \sum_{j=1}^{5}\sum_{i=1}^{5} c_{ij}x_{(10+i)}x_{(10+j)} + 2\sum_{j=1}^{5} d_j x_{(10+j)}^3 - \sum_{i=1}^{10} b_i x_i$$

subject to:

$$g_j(\mathbf{x}) = -2\sum_{i=1}^{5} c_{ij}x_{(10+i)} - 3d_j x_{(10+j)}^2 - e_j + \sum_{i=1}^{10} a_{ij}x_i \le 0,$$
$$j = 1, \ldots, 5$$
$$x_1, x_2, x_4, x_6, \ldots, x_9, x_{11}, x_{13}, x_{14} \in [0,10]$$
$$x_3, x_5, x_{10}, x_{12}, x_{15} \in \{0,1,\ldots,10\}$$

where $\mathbf{b}$ = [−40, −2, −0.25, −4, −4, −1, −40, −60, 5, 1]. The other data can be found in Table S-I. The best known optimal solution is $\mathbf{x}^*$= (x_1, x_2, x_4, x_6, . . . , x_9, x_{11}, x_{13}, x_{14}) = (0, 0, 0, 9.99999999, 0, 0, 2.96750117, 0.39963905, 0.82151768, 0.64848398), and $\mathbf{y}^*$= (x_3, x_5, x_{10}, x_{12}, x_{15}) = (2, 4, 0, 0, 1), and $f(\mathbf{x}^*, \mathbf{y}^*)$ = 41.7399.

$$\mathbf{F13}: \text{minimize: } f(\mathbf{x},\mathbf{y}) = f_1(y_1) + f_2(x_1)$$

where:

$$f_1(y_1) = \begin{cases} 30y_1, & 0 \le y_1 \le 300 \\ 31y_1, & 300 \le y_1 \le 400 \end{cases}$$
$$f_2(x_1) = \begin{cases} 28x_1, & 0 \le x_1 \le 100 \\ 29x_1, & 100 \le x_1 \le 200 \\ 30x_1, & 200 \le x_1 \le 1000 \end{cases}$$

subject to:

$$h_1(\mathbf{x},\mathbf{y}) = -y_1 + 300 - \frac{y_2 x_2}{131.078}\cos(1.48477 - x_4) + \frac{0.90798 y_2^2}{131.078}\cos(1.47588) = 0$$
$$h_2(\mathbf{x},\mathbf{y}) = -x_1 - \frac{y_2 x_2}{131.078}\cos(1.48477 + x_4) + \frac{0.90798 x_2^2 131.078}{\cos}(1.47588) = 0$$
$$h_3(\mathbf{x},\mathbf{y}) = -x_3 - \frac{y_2 x_2}{131.078}\sin(1.48477 + x_4) + \frac{0.90798 x_2^2}{131.078}\sin(1.47588) = 0$$
$$h_4(\mathbf{x},\mathbf{y}) = 200 - \frac{y_2 x_2}{131.078}\sin(1.48477 - x_4) + \frac{0.90798 y_2^2}{131.078}\sin(1.47588) = 0$$
$$x_1 \in [0,1000], x_2 \in [340,420]$$
$$x_3 \in [-1000,1000], x_4 \in [0,0.5236]$$
$$y_1 \in \{0,20,\ldots,400\}, y_2 \in \{340,360,\ldots,420\}$$

The best known optimal solution is $\mathbf{x}^*$= (81.57454322, 416.85149297, −9.77394390, 0.05912763) and $\mathbf{y}^*$= (220, 380), and $f(\mathbf{x}^*, \mathbf{y}^*)$ = 8884.0872.

Table S-I. Data for problems F11 and F12

j	1	2	3	4	5
e_j	-15	-27	-36	-18	-12
c_{1j}	30	-20	-10	32	-10
c_{2j}	-20	39	-6	-31	32
c_{3j}	-10	-6	10	-6	-10
c_{4j}	32	-31	-6	39	-20
c_{5j}	-10	32	-10	-20	30
d_j	4	8	10	6	2
a_{1j}	-16	2	0	1	0
a_{2j}	0	-2	0	0.4	2
a_{3j}	-3.5	0	2	0	0
a_{4j}	0	-2	0	-4	-1
a_{5j}	0	-9	-2	1	-2.8
a_{6j}	2	0	-4	0	0
a_{7j}	-1	-1	-1	-1	-1
a_{8j}	-1	-2	-3	-2	-1
a_{9j}	1	2	3	4	5
a_{10j}	1	1	1	1	1

F14 : minimize: $f(\mathbf{x},\mathbf{y}) = f_1(y_1) + f_2(x_1)$

where:

$$f_1\ (y_1) = \begin{cases} 30y_1, & 0 \leq\ y_1 \leq 300 \\ 31y_1, & 300 \leq\ y_1 \leq 400 \end{cases}$$

$$f_2\ (x_1) = \begin{cases} 28x_1, & 0 \leq\ x_1 \leq 100 \\ 29x_1, & 100 \leq\ x_1 \leq 200 \\ 30x_1, & 200 \leq\ x_1 \leq 1000 \end{cases}$$

subject to:

$$h_1\,(\mathbf{x},\mathbf{y}) = -y_1 + 300 - \frac{y_2x_2}{131.078}\cos\left(1.48477 - x_4\right) + \frac{0.90798y_2^2}{131.078}\cos\left(1.47588\right) = 0$$

$$h_2\,(\mathbf{x},\mathbf{y}) = -x_1 - \frac{y_2x_2}{131.078}\cos\left(1.48477 + x_4\right) + \frac{0.90798x_2^2}{131.078}\cos\left(1.47588\right) = 0$$

$$h_3\,(\mathbf{x},\mathbf{y}) = -x_3 - \frac{y_2x_2}{131.078}\sin\left(1.48477 + x_4\right) + \frac{0.90798x_2^2}{131.078}\sin\left(1.47588\right) = 0$$

$$
\begin{aligned}
h_4(\mathbf{x},\mathbf{y}) =& 200 - \frac{y_2 x_2}{131.078}\sin(1.48477 - x_4) \\
&+ \frac{0.90798 y_2^2}{131.078}\sin(1.47588) = 0
\end{aligned}
$$

$$
\begin{aligned}
& x_1 \in [0,1000], x_2 \in [340,420] \\
& x_3 \in [-1000,1000], x_4 \in [0,0.5236] \\
& y_1 \in \{0,50,...,400\}, y_2 \in \{350,400\}
\end{aligned}
$$

The best known optimal solution is $\mathbf{x}^*$= (51.69905661, 394.30118556, 20.47601024, 0.03816719) and $\mathbf{y}^*$= (250, 350), and $f(\mathbf{x}^*, \mathbf{y}^*)$ = 8947.5736.

F15 : minimize : $f(\mathbf{x},\mathbf{y}) = x_1^2 + y_1^2 + x_1 y_1 - 14x_1 - 16y_1 + (y_2 - 10)^2 + 4(x_2 - 5)^2 + (x_3 - 3)^2 + 2(x_4 - 1)^2 + 5x_5^2 + 7(x_6 - 11)^2 + 2(y_3 - 10)^2 + (x_7 - 7)^2 + 45$

subject to:

$$
\begin{aligned}
& g_1(\mathbf{x},\mathbf{y}) = -105 + 4x_1 + 5y_1 - 3x_5 + 9x_6 \leq 0 \\
& g_2(\mathbf{x},\mathbf{y}) = 10x_1 - 8y_1 - 17x_5 + 2x_6 \leq 0 \\
& g_3(\mathbf{x},\mathbf{y}) = -8x_1 + 2y_1 + 5y_3 - 2x_7 - 12 \leq 0 \\
& g_4(\mathbf{x},\mathbf{y}) = 3(x_1 - 2)^2 + 4(y_1 - 3)^2 + 2y_2^2 - 7x_2 - 120 \leq 0 \\
& g_5(\mathbf{x},\mathbf{y}) = 5x_1^2 + 8y_1 + (y_2 - 6)^2 - 2x_2 - 40 \leq 0 \\
& g_6(\mathbf{x},\mathbf{y}) = x_1^2 + 2(y_1 - 2)^2 - 2x_1 y_1 + 14x_3 - 6x_4 \leq 0 \\
& g_7(\mathbf{x},\mathbf{y}) = 0.5(x_1 - 8)^2 + 2(y_1 - 4)^2 + 3x_3^2 - x_4^2 - 30 \leq 0 \\
& g_8(\mathbf{x},\mathbf{y}) = -3x_1 + 6y_1 + 12(y_3 - 8)^2 - 7x_7 \leq 0 \\
& x_1, x_2, ..., x_7 \in [-10, 10] \\
& y_1, y_2, y_3 \in \{-10, -9, ..., 10\}
\end{aligned}
$$

The best known optimal solution is $\mathbf{x}^*$= (2.45799944, 5.10440319, 0.89287364, 1.45166575, 1.68117614, 9.99999999, 8.66800226) and $\mathbf{y}^*$= (2, 8, 9), and $f(\mathbf{x}^*, \mathbf{y}^*)$ = 28.3514.

F16 : minimize : $f(\mathbf{x},\mathbf{y}) = x_1^2 + y_1^2 + x_1 y_1 - 14x_1 - 16y_1 + (y_2 - 10)^2 + 4(x_2 - 5)^2 + (y_3 - 3)^2 + 2(x_3 - 1)^2 + 5x_4^2 + 7(y_4 - 11)^2 + 2(y_5 - 10)^2 + (x_5 - 7)^2 + 45$

subject to:

$$
\begin{aligned}
& g_1(\mathbf{x},\mathbf{y}) = -105 + 4x_1 + 5y_1 - 3x_4 + 9x_4 \leq 0 \\
& g_2(\mathbf{x},\mathbf{y}) = 10x_1 - 8y_1 - 17x_4 + 2y_4 \leq 0 \\
& g_3(\mathbf{x},\mathbf{y}) = -8x_1 + 2y_1 + 5y_5 - 2x_5 - 12 \leq 0 \\
& g_4(\mathbf{x},\mathbf{y}) = 3(x_1 - 2)^2 + 4(y_1 - 3)^2 + 2y_2^2 - 7x_2 - 120 \leq 0 \\
& g_5(\mathbf{x},\mathbf{y}) = 5x_1^2 + 8y_1 + (y_2 - 6)^2 - 2x_2 - 40 \leq 0 \\
& g_6(\mathbf{x},\mathbf{y}) = x_1^2 + 2(y_1 - 2)^2 - 2x_1 y_1 + 14y_3 - 6x_3 \leq 0 \\
& g_7(\mathbf{x},\mathbf{y}) = 0.5(x_1 - 8)^2 + 2(y_1 - 4)^2 + 3y_3^2 - x_3^2 - 30 \leq 0 \\
& g_8(\mathbf{x},\mathbf{y}) = -3x_1 + 6y_1 + 12(y_5 - 8)^2 - 7x_5 \leq 0 \\
& x_1, x_2, ..., x_5 \in [-10, 10] \\
& y_1, y_2, ..., y_5 \in \{-10, -9, ..., 10\}
\end{aligned}
$$

The best known optimal solution is $\mathbf{x}^*$= (2.45787583, 5.10288399, 1.70160838, 1.68110343, 8.66849668) and $\mathbf{y}^*$= (2, 8, 1, 10, 9), and $f(\mathbf{x}^*, \mathbf{y}^*)$ = 28.4879.

$$
\begin{aligned}
\textbf{F17} : \text{minimize} : f(\mathbf{x},\mathbf{y}) =& 10x_1 - 7x_3 - 18\log(x_2+1) - 19.2\log(x_1 - x_2 + 1) + 5y_4 + 6y_5 \\
& + 8y_6 + 10
\end{aligned}
$$

subject to:

$$
\begin{aligned}
& g_1(\mathbf{x},\mathbf{y}) = -0.8\log(x_2+1) - 0.96\log(x_1 - x_2 + 1) + 0.8x_3 \leq 0 \\
& g_2(\mathbf{x},\mathbf{y}) = x_2 - x_1 \leq 0 \\
& g_3(\mathbf{x},\mathbf{y}) = x_2 - 2y_4 \leq 0 \\
& g_4(\mathbf{x},\mathbf{y}) = x_1 - x_2 - 2y_5 \leq 0 \\
& g_5(\mathbf{x},\mathbf{y}) = -\log(x_2+1) - 1.2\log(x_1 - x_2 + 1) + x_3 + 2y_6 - 2 \leq 0 \\
& g_6(\mathbf{x},\mathbf{y}) = y_4 + y_5 - 1 \leq 0 \\
& x_1, x_2 \in [0,2], x_3 \in [0,1] \\
& y_4, y_5, y_6 \in \{0,1\}
\end{aligned}
$$

The best known optimal solution is $\mathbf{x}^*$= (1.3010, 0, 1) and $\mathbf{y}^*$= (0, 1, 0), and $f(\mathbf{x}^*, \mathbf{y}^*)$ = 6.0098.

$$
\begin{aligned}
\textbf{F18} : \text{minimize} : f(\mathbf{x},\mathbf{y}) =& -0.29x_1 - 0.43x_2 - 0.015x_3 - 0.985x_4 - 0.165x_5 - 0.105x_6 \\
& - 0.37x_7 - 0.2x_8 - 0.49x_9 - 0.34x_{10} - 0.175y_{11} - 0.39y_{12} - 0.83y_{13} \\
& - 0.805y_{14} - 0.06y_{15} - 0.4y_{16} - 0.52y_{17} - 0.415y_{18} - 0.655y_{19} - 0.63y_{20}
\end{aligned}
$$

subject to:

$$
\begin{aligned}
& g_1(\mathbf{x},\mathbf{y}) = \sqrt{0.0001 + \sum_{i=1}^{10} x_i^2 + \sum_{j=11}^{20} y_j^2} - 10 \leq 0 \\
& x_1, x_2, \cdots, x_{10} \in [0,5], y_{11}, y_{12}, \cdots, y_{20} \in \{0,1,\cdots,5\}
\end{aligned}
$$

The best known optimal solution is $\mathbf{x}^*$= (1.2381, 1.8357, 0.0640, 4.2057, 0.7045, 0.4482, 1.5800, 0.8537, 2.0920, 1.4515) and $\mathbf{y}^*$= (1, 2, 4, 4, 0, 2, 2, 2, 3, 3), and $f(\mathbf{x}^*, \mathbf{y}^*)$ = -21.7491.

$$
\textbf{F19} : \text{minimize} : f(\mathbf{x},\mathbf{y}) = 7.5y_3 + 5.5y_4 + 7x_5 + 6x_6 + 5(x_1 + x_2)
$$

subject to:

$$
\begin{aligned}
& h_1(\mathbf{x},\mathbf{y}) = y_3 + y_4 - 1 = 0 \\
& h_2(\mathbf{x},\mathbf{y}) = 0.9(1 - e^{-0.5x_5})x_1 + 0.8(1 - e^{-0.4x_6})x_2 = 10 \\
& h_3(\mathbf{x},\mathbf{y}) = 0.9(1 - e^{-0.5x_5})x_1 y_3 + 0.8(1 - e^{-0.4x_6})x_2 y_4 = 10 \\
& g_1(\mathbf{x},\mathbf{y}) = x_1 - 20x_3 \leq 0 \\
& g_2(\mathbf{x},\mathbf{y}) = x_2 - 20x_4 \leq 0 \\
& g_3(\mathbf{x},\mathbf{y}) = x_5 - 10x_3 \leq 0 \\
& g_4(\mathbf{x},\mathbf{y}) = x_6 - 10x_4 \leq 0 \\
& g_5(\mathbf{x},\mathbf{y}) = -0.9(1 - e^{-0.5x_5})x_1 \leq 0 \\
& g_6(\mathbf{x},\mathbf{y}) = -0.8(1 - e^{-0.4x_6})x_2 \leq 0 \\
& x_1, x_5, x_6 \in [0,100], x_2 \in [0,50] \\
& y_3, y_4 \in \{0,1\}
\end{aligned}
$$

The best known optimal solution is $\mathbf{x}^*$= (13.4279, 0, 3.5142, 0) and $\mathbf{y}^*$= (1, 0), and $f(\mathbf{x}^*, \mathbf{y}^*)$ = 99.2390.

F20 : minimize : $f(x,\mathbf{y}) = -0.00201x_1^2y_2^4y_3$

subject to:

$$g_1(x,\mathbf{y}) = y_2^2y_3 - 675 \leq 0$$
$$g_2(x,\mathbf{y}) = 0.1x_1^2y_2^2 - 0.419 \leq 0$$
$$x_1 \in [0.1, 0.2]$$
$$y_2, y_3 \in \{1, 2, \cdots, 200\}$$

The best known optimal solution is $\mathbf{x}^*$= 0.1365 and $\mathbf{y}^*$= (15, 3), and $f(\mathbf{x}^*, \mathbf{y}^*)$ = -5.6848.

F21 : minimize : $f(\mathbf{x},\mathbf{y}) = 5.3578547x_1^2 + 0.8356891x_3y_4 + 37.293239y_4 - 40792.141$

subject to:

$$g_1(\mathbf{x},\mathbf{y}) = -(85.334407 - 0.0022053x_1x_3 + 0.0056858x_3y_5 + 0.0006262x_2y_4) \leq 0$$
$$g_2(\mathbf{x},\mathbf{y}) = -(80.51249 + 0.0021813x_1^2 + 0.0071317x_3y_5 + 0.0029955y_4y_5 - 90) \leq 0$$
$$g_3(\mathbf{x},\mathbf{y}) = -(9.300961 + 0.0019085x_1x_2 + 0.0047026x_1x_3 + 0.0012547x_1y_4 - 20) \leq 0$$
$$g_4(\mathbf{x},\mathbf{y}) = 85.334407 - 0.0022053x_1x_3 + 0.0056858x_3y_5 + 0.0006262x_2y_4 - 92 \leq 0$$
$$g_5(\mathbf{x},\mathbf{y}) = 80.51249 + 0.0021813x_1^2 + 0.0071317x_3y_5 + 0.0029955y_4y_5 - 110 \leq 0$$
$$g_6(\mathbf{x},\mathbf{y}) = 9.300961 + 0.0019085x_1x_2 + 0.0047026x_1x_3 + 0.0012547x_1y_4 - 25 \leq 0$$
$$x_1, x_2, x_3 \in [27, 45]$$
$$y_4 \in \{78, 79, \cdots, 102\}$$
$$y_5 \in \{33, 34, \cdots, 45\}$$

The best known optimal solution is $\mathbf{x}^*$= (29.9953, 45, 36.7758) and $\mathbf{y}^*$= (78, 33), and $f(\mathbf{x}^*, \mathbf{y}^*)$ = -30665.5387.

F22 : minimize : $f(\mathbf{x},\mathbf{y}) = -\sum_{i=1}^{3} x_i + 5\sum_{i=4}^{7}\left(y_i - y_i^2\right) - \sum_{i=8}^{13} y_i$

subject to:

$$g_1(\mathbf{x},\mathbf{y}) = x_1 + x_2 + 2y_4 + 2y_5 - 10 \leq 0$$
$$g_2(\mathbf{x},\mathbf{y}) = x_1 + x_3 + 2y_4 + 2y_6 - 10 \leq 0$$
$$g_3(\mathbf{x},\mathbf{y}) = x_2 + x_3 + 2y_5 + 2y_6 - 10 \leq 0$$
$$g_4(\mathbf{x},\mathbf{y}) = x_1 - 8y_4 \leq 0$$
$$g_5(\mathbf{x},\mathbf{y}) = x_2 - 8y_5 \leq 0$$
$$g_6(\mathbf{x},\mathbf{y}) = x_3 - 8y_6 \leq 0$$
$$g_7(\mathbf{x},\mathbf{y}) = x_1 - 2y_7 - y_8 \leq 0$$
$$g_8(\mathbf{x},\mathbf{y}) = x_2 - 2y_9 - y_{10} \leq 0$$
$$g_9(\mathbf{x},\mathbf{y}) = x_3 - 2y_{11} - y_{12} \leq 0$$
$$x_1, x_2, x_3 \in [0, 100]$$
$$y_4, y_5, \ldots, y_{13} \in \{0, 1\}$$

The best known optimal solution is $\mathbf{x}^*$= (3, 3, 3) and $\mathbf{y}^*$= (1, 1, 1, 1, 1, 1, 1, 1, 1, 1), and $f(\mathbf{x}^*, \mathbf{y}^*)$ = -15.

F23 : minimize : $f(\mathbf{x},\mathbf{y}) = (x_1-1)^2 + (x_2-2)^2 + (x_3-3)^2 + (y_4-1)^2 + (y_5-1)^2 + (y_6-1)^2 - \log(y_7+1)$

subject to:

$$
\begin{aligned}
&g_1(\mathbf{x},\mathbf{y}) = x_2 + y_5 - 1.8 \leq 0\\
&g_2(\mathbf{x},\mathbf{y}) = x_2^2 + y_5^2 - 1.64 \leq 0\\
&g_3(\mathbf{x},\mathbf{y}) = x_1 + x_2 + x_3 + y_4 + y_5 + y_6 - 5 \leq 0\\
&g_4(\mathbf{x},\mathbf{y}) = x_3 + y_6 - 2.5 \leq 0\\
&g_5(\mathbf{x},\mathbf{y}) = x_3^2 + y_6^2 - 4.25 \leq 0\\
&g_6(\mathbf{x},\mathbf{y}) = x_1^2 + x_2^2 + x_3^2 + y_6^2 - 5.5 \leq 0\\
&g_7(\mathbf{x},\mathbf{y}) = x_1 + y_7 - 1.2 \leq 0\\
&g_8(\mathbf{x},\mathbf{y}) = x_3^2 + y_5^2 - 4.64 \leq 0\\
&g_9(\mathbf{x},\mathbf{y}) = x_1 + y_4 - 1.2 \leq 0\\
&x_1, x_2, x_3 \in [0, 1 \times 10^6]\\
&y_4, y_5, y_6, y_7 \in \{0, 1\}
\end{aligned}
$$

The best known optimal solution is $\mathbf{x}^*$= (0.2, 1.2806, 1.9545) and $\mathbf{y}^*$= (1, 0, 0, 1), and $f(\mathbf{x}^*, \mathbf{y}^*)$ = 3.5575.

F24 : minimize : $f(\mathbf{x},\mathbf{y}) = 3(x_1 - 11)^2 + 10x_2^6 + 7x_3^2 + x_4^4 - 4x_3x_4 - 10x_3 - 8x_4 + (y_5 - 10)^2 + 5(y_6 - 12)^2 + x_7^4$

subject to:

$$
\begin{aligned}
&g_1(\mathbf{x},\mathbf{y}) = 4x_1^2 + 5x_2 + 2y_5^2 + 3y_6^4 + y_7 - 127 \leq 0\\
&g_2(\mathbf{x},\mathbf{y}) = x_1 - x_2 + 7y_5 + 3y_6 + 10y_7^2 - 282 \leq 0\\
&g_3(\mathbf{x},\mathbf{y}) = 6x_3^2 - 8x_4 + 23y_5 + y_6^2 - 196 \leq 0\\
&g_4(\mathbf{x},\mathbf{y}) = 5x_3 - 11y_4 + 4y_5^2 + y_6^2 - 3y_5y_6 + 2y_7^2 \leq 0\\
&x_1, x_2, x_3, x_4 \in [-10, 10]\\
&y_5, y_6, y_7 \in \{-10, -9, \ldots, 10\}
\end{aligned}
$$

The best known optimal solution is $\mathbf{x}^*$= (4.3339, -0.6260, 1.1323, 1.4632) and $\mathbf{y}^*$= (2, 2, -1), and $f(\mathbf{x}^*, \mathbf{y}^*)$ = 682.8160.

F25 : minimize : $f(\mathbf{x},\mathbf{y}) = 0.6224x_1x_2y_3 + 1.7781x_1^2y_4 + 3.1661x_2y_3^2 + 19.84x_1y_3^2$

subject to:

$$
\begin{aligned}
&g_1(\mathbf{x},\mathbf{y}) = 0.0193x_1 - y_3 \leq 0\\
&g_2(\mathbf{x},\mathbf{y}) = 0.00954x_1 - y_4 \leq 0\\
&g_3(\mathbf{x},\mathbf{y}) = 1296000 - \frac{4}{3}\pi x_1^3 - \pi x_1^2 x_2 = 0\\
&x_1, x_2 \in [10, 221.2]\\
&y_3, y_4 \in \{0.0625, 0.1250, ..., 6.1875\}
\end{aligned}
$$

The best known optimal solution is $\mathbf{x}^*$= (42.0984 176.6366) and $\mathbf{y}^*$= (0.8125, 0.4375), and $f(\mathbf{x}^*, \mathbf{y}^*)$ = 6059.7143.

F26 : minimize : $f(\mathbf{x},\mathbf{y}) = \left(0.14427932477276 - \frac{x_1 \cdot x_2}{x_3 \cdot x_4}\right)^2$

subject to:

$$h_1(\mathbf{x},\mathbf{y}) = x_1 - y_5 = 0$$

$$h_2(\mathbf{x},\mathbf{y}) = x_2 - y_6 = 0$$
$$h_3(\mathbf{x},\mathbf{y}) = x_3 - y_7 = 0$$
$$h_4(\mathbf{x},\mathbf{y}) = x_4 - y_8 = 0$$
$$x_1, x_2, x_3, x_4 \in [12, 60]$$
$$y_5, y_6, y_7, y_8 \in \{12, 60\}$$

The best known optimal solution is $\mathbf{x}^*$= (17, 15, 31, 57) and $\mathbf{y}^*$= (17, 15, 31, 57), and $f(\mathbf{x}^*, \mathbf{y}^*)$ = 0.

F27 : minimize : $f(\mathbf{x},\mathbf{y}) = 5.9207859 + 9.99999999999999 \times 10^{-05} \times (5.3578547 y_3^2 + 0.8356891 y_1 y_5 + 37.293239 y_1)$

subject to:

$$h_1(\mathbf{x},\mathbf{y}) = -(0.0056858 y_2 y_5 + 0.0006262 y_1 y_4 - 0.0022053 y_3 y_5) + x_6 - 85.334407$$
$$h_2(\mathbf{x},\mathbf{y}) = -(0.0071317 y_2 y_5 + 0.0029955 y_1 y_2 + 0.0021813 y_3^2) + x_7 - 80.51249$$
$$h_3(\mathbf{x},\mathbf{y}) = -(0.0047026 y_3 y_5 + 0.0012547 y_1 y_3 + 0.0019085 y_3 y_4) + x_8 - 9.300961$$
$$y_1, y_2, y_3, y_4, y_5 \in \{1, 200\}$$
$$x_6 \in [0, 92],\ x_7 \in [90, 110],\ x_8 \in [20, 25]$$

The best known optimal solution is $\mathbf{x}^*$= (90.8476, 91.7561, 20.0271) and $\mathbf{y}^*$= (1, 9, 9, 200, 172), and $f(\mathbf{x}^*, \mathbf{y}^*)$ = 5.9823.

F28 : minimize : $f(\mathbf{x},\mathbf{y}) = 1.10471 y_3^2 y_4 + 0.04811 y_1 y_2 (14 + y_4)$

subject to:

$$h_1(\mathbf{x},\mathbf{y}) = -\frac{4243.28147100424}{y_3 y_4} + x_5 = 0$$
$$h_2(\mathbf{x},\mathbf{y}) = -\sqrt{0.25 y_4^2 + (0.5 y_1 + 0.5 y_3)^2} + x_7 = 0$$
$$h_3(\mathbf{x},\mathbf{y}) = -\frac{(59405.9405940594 + 2121.64073550212 y_4) x_7}{y_3 y_4 (0.0833333333333333 y_4^2 + (0.5 y_1 + 0.5 y_3)^2)} + x_6 = 0$$
$$h_4(\mathbf{x},\mathbf{y}) = -0.5 \frac{y_4}{x_7} + x_8 = 0$$
$$g_1(\mathbf{x},\mathbf{y}) = \sqrt{x_5^2 + 2 x_5 x_6 x_8 + x_6^2} - 13600 \leq 0$$
$$g_2(\mathbf{x},\mathbf{y}) = \frac{504000}{y_1^2 y_2} - 30000 \leq 0$$
$$g_3(\mathbf{x},\mathbf{y}) = -y_2 + y_3 \leq 0$$
$$g_4(\mathbf{x},\mathbf{y}) = -0.0204744897959184 \sqrt{1 \times 10^{13} y_2^3 y_1^2 y_2^3} \times (1 - 0.0282346219657891 y_1) + 6000 \leq 0$$
$$g_5(\mathbf{x},\mathbf{y}) = \frac{2.1952}{y_1^3} - 0.25 \leq 0$$
$$y_1, y_2 \in \{1, 200\}$$
$$y_3, y_4 \in \{1, 20\}$$
$$x_5, x_6, x_7, x_8 \in [0, 1 \times 10^5]$$

The best known optimal solution is $\mathbf{x}^*$= (2121.6408, 10782.9331, 3.1624, 0.3162) and $\mathbf{y}^*$= (5, 1, 1, 2), and $f(\mathbf{x}^*, \mathbf{y}^*)$ = 6.0582.

S-II. ADDITIONAL RESULTS

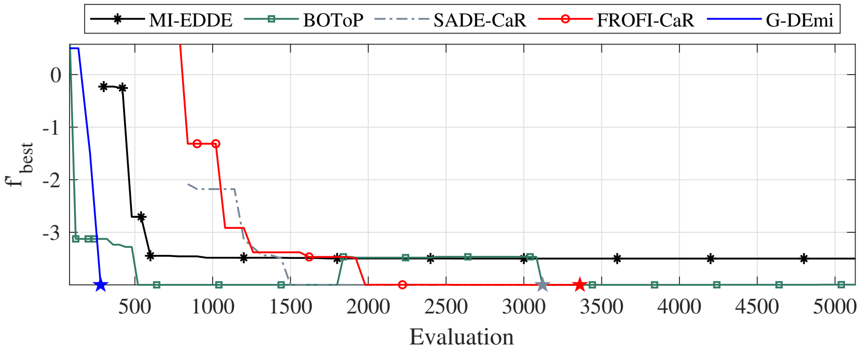

Figure S-I. Convergence curves of the median solutions from the 30 runs for problem F3.

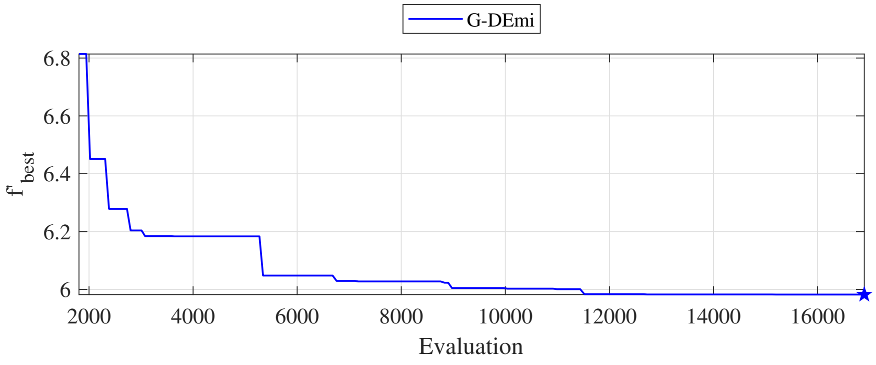

Figure S-II. Convergence curves of the median solutions from the 30 runs for problem F27. No other algorithm reached feasible solutions in the range of evaluations presented.

Table S-II. Average execution times in seconds and the average value of the objective function provided by G-DEmi on the F3 problem.

		$k_{max} = 0$	$k_{max} = 25$	$k_{max} = 50$	$k_{max} = 100$
$T_{min} = 1 \times 10^{-3}$	Ave.	-3.5833	**-4.0000**	**-4.0000**	**-4.0000**
	Time	3.4434	3.4672	3.3791	3.6942
$T_{min} = 1 \times 10^{-30}$	Ave.	-3.5833	**-4.0000**	**-4.0000**	**-4.0000**
	Time	3.3812	3.6974	3.6593	3.9220
$T_{min} = 1 \times 10^{-60}$	Ave.	-3.5667	**-4.0000**	**-4.0000**	**-4.0000**
	Time	3.4050	3.5872	3.5783	3.9204
$T_{min} = 1 \times 10^{-80}$	Ave.	-3.5500	**-4.0000**	**-4.0000**	**-4.0000**
	Time	3.4597	3.5580	3.5902	3.9860

Table S-III. Average execution times in seconds and the average value of the objective function provided by G-DEmi on the F12 problem.

		$k_{max} = 0$	$k_{max} = 25$	$k_{max} = 50$	$k_{max} = 100$
$T_{min} = 1 \times 10^{-3}$	Ave.	45.5244	**41.7399**	41.7450	41.7515
	Time	5.3546	23.1976	30.7741	43.9094
$T_{min} = 1 \times 10^{-30}$	Ave.	56.3351	**41.7399**	41.7450	**41.7399**
	Time	5.4118	28.6635	42.6500	69.8246
$T_{min} = 1 \times 10^{-60}$	Ave.	49.3507	41.7450	**41.7399**	**41.7399**
	Time	5.4309	28.1685	43.2747	73.8161
$T_{min} = 1 \times 10^{-80}$	Ave.	49.1143	41.7632	**41.7399**	**41.7399**
	Time	5.4355	27.0197	43.2668	71.2409

Table S-IV. Average execution times in seconds and the average value of the objective function provided by G-DEmi on the F27 problem.

		$k_{max} = 0$	$k_{max} = 25$	$k_{max} = 50$	$k_{max} = 100$
$T_{min} = 1 \times 10^{-3}$	Ave.	NA	5.9826	**5.9823**	5.9832
	Time	5.8850	6.8628	6.7666	6.8239
$T_{min} = 1 \times 10^{-30}$	Ave.	NA	**5.9823**	**5.9823**	**5.9823**
	Time	5.8674	5.8369	5.8406	6.0280
$T_{min} = 1 \times 10^{-60}$	Ave.	NA	**5.9823**	**5.9823**	5.9851
	Time	5.8153	5.8915	6.0963	6.5128
$T_{min} = 1 \times 10^{-80}$	Ave.	NA	**5.9823**	**5.9823**	**5.9823**
	Time	6.1649	5.8499	6.0117	6.3620

Table S-V. Performance comparison conducted over 30 independent runs with 200,000 objective function evaluations for PSOmv, EDAmv, and G-DEmi (Part 1 of 2).

Problem	Parameter	PSOmv	EDAmv	G-DEmi
F1	FR	**100**	**100**	**100**
	SR	0	96.67	**100**
	Ave ± Std	1.70E+01±0.00E+00 +	1.31E+01±7.30E-01 ≈	1.30E+01±7.23E-15
	T	4.83E+00	2.56E-01	3.60E+00
F2	FR	**100**	**100**	**100**
	SR	6.67	**100**	**100**
	Ave ± Std	2.87E+00±2.13E+00 +	1.00E+00±0.00E+00 ≈	1.00E+00±0.00E+00
	T	3.59E+01	5.05E-01	3.67E+00
F3	FR	**100**	**100**	**100**
	SR	0	**100**	**100**
	Ave ± Std	-3.57E+00±1.72E-01 +	-4.00E+00±0.00E+00 ≈	-4.00E+00±0.00E+00
	T	2.08E+01	3.92E-01	3.72E+00
F4	FR	**100**	**100**	**100**
	SR	**100**	**100**	**100**
	Ave ± Std	-6.00E+00±0.00E+00 ≈	-6.00E+00±0.00E+00 ≈	-6.00E+00±0.00E+00
	T	2.08E+01	3.81E-01	3.43E+00
F5	FR	**100**	**100**	**100**
	SR	6.67	67	**100**
	Ave ± Std	1.17E+00±2.51E-01 +	5.80E-01±4.75E-01 +	2.50E-01±2.54E-05
	T	7.71E+00	2.42E-01	3.48E+00
F6	FR	**100**	**100**	**100**
	SR	47	**100**	**100**
	Ave ± Std	-6.10E+03±7.47E+02 +	-6.78E+03±2.78E-12 ≈	-6.78E+03±2.78E-12
	T	2.07E+01	4.39E-01	3.60E+00
F7	FR	93.33	**100**	**100**
	SR	0	33.33	**100**
	Ave ± Std	NA	6.77E-01±3.61E-01 +	2.11E-01±1.13E-16
	T	1.80E+01	4.88E-01	7.45E+00
F8	FR	66.67	63.33	**100**
	SR	0	0	**97**
	Ave ± Std	NA	NA	7.06E+03±6.90E+00
	T	1.11E+02	1.21E+00	1.52E+01
F9	FR	36.67	56.67	**100**
	SR	0	0	**100**
	Ave ± Std	NA	NA	7.08E+03±0.00E+00
	T	6.23E+01	1.03E+00	8.72E+00
F10	FR	6.67	60	**100**
	SR	0	0	**100**
	Ave ± Std	NA	NA	7.13E+03±0.00E+00
	T	5.13E+01	9.20E-01	8.12E+00
F11	FR	100	100	**100**
	SR	0	0	**60**
	Ave ± Std	9.60E+01±6.64E+01 +	4.27E+01±6.82E+00 +	3.35E+01±4.95E-02
	T	2.82E+01	1.31E+00	5.49E+01
F12	FR	**100**	**100**	**100**
	SR	0	0	**100**
	Ave ± Std	1.98E+02±8.31E+01 +	9.11E+01±2.87E+01 +	4.17E+01±3.69E-13
	T	4.59E+01	1.25E+00	4.57E+01
F13	FR	**100**	**100**	**100**
	SR	0	0	**100**
	Ave ± Std	9.02E+03±9.72E+01 +	8.98E+03±8.16E+01 +	8.88E+03±5.47E-12
	T	2.12E+01	5.95E-01	1.60E+01
F14	FR	**100**	**100**	**100**
	SR	0	50	**100**
	Ave ± Std	9.12E+03±8.40E+01 +	8.98E+03±8.07E+01 +	8.95E+03±3.70E-12
	T	1.82E+01	5.91E-01	6.87E+00
F15	FR	**100**	**100**	**100**
	SR	0	0	**96.67**
	Ave ± Std	4.29E+01±9.54E+00 +	3.38E+01±3.42E+00 +	2.84E+01±7.53E-02
	T	3.25E+01	7.75E-01	1.16E+01

Table S-VI. Performance comparison conducted over 30 independent runs with 200,000 objective function evaluations for PSOmv, EDAmv, and G-DEmi (Part 2 of 2).

Problem	Parameter	PSOmv	EDAmv	G-DEmi
F16	FR	**100**	**100**	**100**
	SR	0	0	**100**
	Ave ± Std	6.84E+01±4.21E+01 +	4.44E+01±1.41E+01 +	2.85E+01±9.67E-15
	T	5.23E+01	7.23E-01	9.60E+00
F17	FR	0	36.67	**100**
	SR	0	0	**100**
	Ave ± Std	NA	NA	6.01E+00±2.71E-15
	T	2.34E+01	5.60E-01	5.04E+00
F18	FR	**100**	**100**	**100**
	SR	0	57	**100**
	Ave ± Std	-2.17E+01±2.86E-02 +	-2.17E+01±1.21E-02 +	-2.17E+01±1.72E-08
	T	7.91E+01	1.26E+00	1.51E+01
F19	FR	0	0	**100**
	SR	0	0	**100**
	Ave ± Std	NA	NA	9.92E+01±0.00E+00
	T	1.60E+01	5.81E-01	8.17E+00
F20	FR	**100**	**100**	**100**
	SR	0	43.33	**100**
	Ave ± Std	-4.87E+00±4.06E-01 +	-5.24E+00±4.24E-01 +	-5.68E+00±9.03E-16
	T	4.87E+01	6.13E-01	3.85E+00
F21	FR	**100**	**100**	**100**
	SR	93.33	**100**	**100**
	Ave ± Std	-3.07E+04±1.24E+01 ≈	-3.07E+04±1.89E-09 ≈	-3.07E+04±1.44E-11
	T	2.23E+01	4.88E-01	4.43E+00
F22	FR	**100**	**100**	**100**
	SR	**100**	**100**	**100**
	Ave ± Std	-1.50E+01±0.00E+00 ≈	-1.50E+01±1.12E-09 ≈	-1.50E+01±0.00E+00
	T	7.25E+01	7.21E-01	4.10E+00
F23	FR	**100**	**100**	**100**
	SR	23.33	10	**93.33**
	Ave ± Std	4.24E+00±5.39E-01 +	4.49E+00±4.27E-01 +	3.56E+00±5.61E-03
	T	3.03E+01	4.95E-01	5.00E+00
F24	FR	**100**	**100**	**100**
	SR	20	60	**100**
	Ave ± Std	6.85E+02±4.16E+00 +	6.83E+02±1.87E+00 +	6.83E+02±6.68E-14
	T	3.38E+01	6.60E-01	6.22E+00
F25	FR	**100**	60	**100**
	SR	0	26.67	**100**
	Ave ± Std	8.25E+03±1.01E+03 +	NA	6.06E+03±9.25E-13
	T	4.47E+01	6.40E-01	4.20E+00
F26	FR	**100**	**100**	**100**
	SR	0	13.33	**100**
	Ave ± Std	7.84E-01±2.72E-01 +	4.54E-02±1.05E-01 +	1.39E-07±2.71E-07
	T	6.03E+01	8.80E-01	5.68E+00
F27	FR	**100**	**100**	**100**
	SR	0	0	**100**
	Ave ± Std	8.25E+00±5.33E-01 +	7.55E+00±5.61E-01 +	5.98E+00±0.00E+00
	T	1.44E+02	1.26E+00	5.70E+00
F28	FR	**100**	**100**	**100**
	SR	0	0	**100**
	Ave ± Std	6.38E+03±2.10E+03 +	7.35E+03±2.66E+03 +	6.06E+00±2.71E-15
	T	9.00E+01	1.18E+00	8.74E+00
Mean FR		85.83	88.45	**100**
Mean SR		14.17	37.74	**98.10**
WRST [+/≈/-]		[25/3/0]	[21/7/0]	

S-III. PARAMETERS OF SUBWAY OPTIMIZATION PROBLEM

Table S-VII. Parameters used in SOP

Parameter	Value
Weight of the train, [lbs]	$W = 78,000$
Effective weight of the train, [lbs]	$W_{eff} = 85,200$
Scaling factor for units, [s/h/ft/mile]	$\gamma = 3600/5280$
Front surface of the train, [mile^2]	$a = 100/(5280)^2$
Drag formula parameter, adim	$b = 0.045$
Drag formula parameter, adim	$c = 0.240306$
Constant braking when coasting, adim	$C = 0.367$
Gravity, [ft/s^2]	$g = 32.2$
Percentage of working machines, adim	$e = 1$
Maximal deceleration, [ft/s^2]	$u_{max} = 4.4$

Table S-VIII. Operation Modes Dependent Parameters.

Parameter	Value
Velocity limits, [mph]	v_1=0.979474
	v_2=6.73211
	v_3=14.2658
Accelerations, [lbs]	a_1=6017.611205
	a_2=12348.34865
	a_3=11124.63729
Energy consumption	p_1=106.1951102
	p_2=180.9758408
	p_3=354.136479

Table S-IX. Coefficients used in SOP

$b_0(1) = -0.1983670410 \times 10^2$	$c_0(1) = 0.3629738340 \times 10^2$
$b_1(1) = 0.1952738055 \times 10^3$	$c_1(1) = -0.2115281047 \times 10^3$
$b_2(1) = 0.2061789974 \times 10^4$	$c_2(1) = 0.7488955419 \times 10^3$
$b_3(1) = -0.7684409308 \times 10^3$	$c_3(1) = -0.9511076467 \times 10^3$
$b_4(1) = 0.2677869201 \times 10^3$	$c_4(1) = 0.5710015123 \times 10^3$
$b_5(1) = -0.3159629687 \times 10^2$	$c_5(1) = -0.1221306465 \times 10^3$
$b_0(2) = -0.1577169936 \times 10^3$	$c_0(2) = 0.4120568887 \times 10^2$
$b_1(2) = 0.3389010339 \times 10^4$	$c_1(2) = 0.3408049202 \times 10^3$
$b_2(2) = 0.6202054610 \times 10^4$	$c_2(2) = -0.1436283271 \times 10^3$
$b_3(2) = -0.4608734450 \times 10^4$	$c_3(2) = 0.8108316584 \times 10^2$
$b_4(2) = 0.2207757061 \times 10^4$	$c_4(2) = -0.5689703073 \times 10^1$
$b_5(2) = -0.3673344160 \times 10^3$	$c_5(2) = -0.2191905731 \times 10^1$

S-IV. TIME COMPLEXITY

As a complement to the reported execution times, the algorithm's complexity is analyzed below. However, this analysis is more of a general estimate than a mathematically accurate formula. Big-O notation provides an upper limit on the algorithm's running time, indicating the number of times each main operation runs. It represents the worst-case complexity of an algorithm with a function $O(f(n))$.

Differential evolution consists of usual operators (mutation, crossover, selection). Its complexity is $O(g(nm+nm+n))$, where g is the number of generations, n is the population size, and m is the variables per individual. The previous analysis reveals that the complexity of mutation and crossover is proportional to g, m, and n. In contrast, the selection process is not affected by the length of the solution vector.

Once the gradient-based repair method is included, the principal operators change to mutation, crossover, selection, and repair. During the repair process, we have two main processes: calculating $\nabla\mathbf{V}$ and computing the inverse of $\nabla\mathbf{V}$. Since the number of variables is often higher than the number of constraints, $\nabla\mathbf{V}$ can be considered of dimensions m. Therefore, the complexity of $\nabla\mathbf{V}$ is $O(m)$, and the complexity of the $\nabla\mathbf{V}$ inversion is $O(m^3)$, making the matrix inversion the dominant operation. The worst-case scenario for the repair process involves addressing all individuals in the population over K_{max} iterations. Since our proposal focuses on MINLP problems, the solution vector includes at least one integer variable. Hence, the repair method involves $n(m-1)^3$ operations repeated K_{max} times, and the complexity is $O(nK_{max}(m-1)^3)$. Then, the overall complexity expression is modified as $O(g(nm+nm+n+nK_{max}(m-1)^3)$. After removing negligible terms, the expression can be reduced to $O(gnm^3K_{max})$.
